# Facile isomerization of silyl enol ethers catalyzed by triflic imide and its application to one-pot isomerization–(2 + 2) cycloaddition

**DOI:** 10.3762/bjoc.8.73

**Published:** 2012-04-27

**Authors:** Kazato Inanaga, Yu Ogawa, Yuuki Nagamoto, Akihiro Daigaku, Hidetoshi Tokuyama, Yoshiji Takemoto, Kiyosei Takasu

**Affiliations:** 1 Graduate School of Pharmaceutical Sciences, Tohoku University, Aobayama, Sendai 980-8578, Japan; 2 Graduate School of Pharmaceutical Sciences, Kyoto University, Yoshida, Sakyo, Kyoto 606-8501, Japan

**Keywords:** isomerization, one-pot reaction, organocatalysis, silyl enol ethers, triflic imide

## Abstract

A triflic imide (Tf_2_NH) catalyzed isomerization of kinetically favourable silyl enol ethers into thermodynamically stable ones was developed. We also demonstrated a one-pot catalytic reaction consisting of (2 + 2) cycloaddition and isomerization. In the reaction sequence, Tf_2_NH catalyzes both of the reactions.

## Introduction

Silyl enol ethers, which are isolable equivalents of metal enolates, are useful and important intermediates in synthetic chemistry [1–3]. They react as a good nucleophile for the introduction of a carbon skeleton or a functional group at the α-position of a carbonyl group under appropriate conditions. Although silyl enol ethers are easily prepared from the corresponding ketones, the regiochemical issue would arise in the case of asymmetric ketones. Treatment with a strong base such as lithium diisopropylamide (LDA), followed by silyl chloride, under cryogenic conditions selectively affords kinetically favourable silyl enol ethers. On the other hand, thermodynamically stable ones can be predominantly obtained by the reaction with a silylating agent in the presence of a weak base, such as triethylamine, under equilibration conditions. Although the preparation of silyl enol ethers has been extensively studied, there have only been a limited number of studies on their isomerization [4–7]. Deyine reported that a catalytic amount of triethylammonium chloride promotes the isomerization to give thermodynamically favourable ones in moderate yield [5]. However, harsh conditions (reaction temperature: ca. 100 to 200 °C) were required for the complete equilibration. Yamamoto and co-workers reported that a SnCl_4_–(BINOL monomethyl ether) complex (5–10 mol %) catalyzes the isomerization of silyl enol ethers at −78 °C [6]. By using this catalyst, they remarkably achieved the kinetic resolution of racemic silyl enol ethers. To make this isomerization synthetically useful and valuable, the development of more-reactive catalysts and a facile procedure would be required. In this communication, we describe isomerization of silyl enol ethers by an organocatalyst under mild conditions and its application to a one-pot catalytic reaction involving isomerization of silyl enol ethers and (2 + 2) cycloaddition.

## Results and Discussion

During our research on triflic imide (Tf_2_NH)-catalyzed reactions [8], we accidentally found that the isomerization of kinetically favourable silyl enol ethers into thermodynamically stable ones occurs smoothly in the presence of Tf_2_NH. When the TBS enol ether **1a** was treated with a catalytic amount of Tf_2_NH (1.0 mol %) in CH_2_Cl_2_ at ambient temperature, isomerization resulted in the thermodynamically stable **2a** in 92% yield along with the recovered **1a** and ketone **3** (Table 1, entry 1). Equilibrium was reached within 5 min. The reaction using 20 mol % of Tf_2_NH resulted in an increase of decomposition into **3** (entry 2). When the reaction was performed at −10 °C, the chemical yield of **2** was slightly improved (entry 3). In contrast, no isomerization was observed at −78 °C even after 1 h (entry 4). The catalytic isomerization reaction also proceeded in toluene (entry 5), but no (or almost no) isomerization occurred in CH_3_CN (entry 6). When 10-camphorsulfonic acid (5 mol %) was used as a catalyst for 1 h, the isomerization was incomplete (entry 7). Enol ethers bearing typical silyl groups were also isomerized (entries 8–12). The decomposition of TMS enol ether **1b** into **3b** slightly increased at ambient temperature compared to that at −10 °C (entries 8 and 9). In the reaction of TIPS enol ether **1d**, the reaction rate decreased and more catalyst (5 mol %) was necessary to achieve equilibrium within 5 min (entry 12).

**Table 1:** Tf_2_NH-catalyzed isomerization of silyl enol ethers.^a,b^

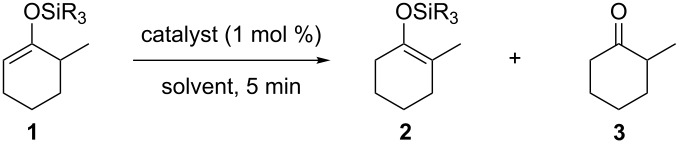						

entry	**1** (SiR_3_)	solvent	temp. (°C)	% yield		

				**2**	**1** (recovd.)	**3**

1	**1a** (TBS)	CH_2_Cl_2_	rt	92	6	2
2^c^	**1a**	CH_2_Cl_2_	rt	71	4	25
3	**1a**	CH_2_Cl_2_	−10	93	4	3
4^d^	**1a**	CH_2_Cl_2_	−78	1	97	2
5	**1a**	toluene	−10	92	5	2
6	**1a**	CH_3_CN	−10	2	96	2
7^d,e,f^	**1a**	CH_2_Cl_2_	−10	25	64	11
8	**1b** (TMS)	CH_2_Cl_2_	rt	85	6	9
9	**1b**	CH_2_Cl_2_	−10	91	4	5
10	**1b**	CH_2_Cl_2_	−78	0	95	5
11	**1c** (TES)	CH_2_Cl_2_	−10	78	5	17
12^e^	**1d** (TIPS)	CH_2_Cl_2_	−10	92	3	5

^a^Yields were determined by GC–MS. ^b^Regioisomer **1** (>99% purity) was used as a substrate. ^c^20 mol % of catalyst was used. ^d^Reactions were carried out for 1 h. ^e^5 mol % of catalyst was used. ^f^10-Camphorsulfonic acid was used as a catalyst.

Several silyl enol ethers were explored for catalytic isomerization under the optimized conditions (1 mol % of Tf_2_NH, −10 °C, CH_2_Cl_2_). The results are summarized in Table 2. All the kinetically favourable silyl enol ethers **1** were smoothly isomerized to the thermodynamically stable **2** in the presence of Tf_2_NH.

**Table 2:** Substrate scope for Tf_2_NH-catalyzed isomerization.^a,b^

entry	substrate	product	% yield of **2**	recovd. **1** (%)

1^c^	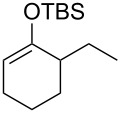 **1e**	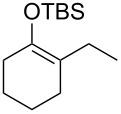 **2e**	83	7
2^d^	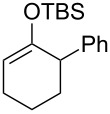 **1f**	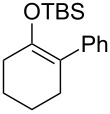 **2f**	89	11
3^e^	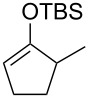 **1g**	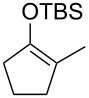 **2g**	95	3
4^f,g^	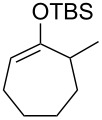 **1h**	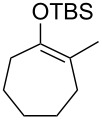 **2h**	99	1

^a^Reactions were performed under the same conditions as given in Table 1, entry 3. ^b^Yields were determined by GC–MS. ^c^Purity of **1e** is 99% (including isomer **2e** (1%)). ^d^Purity of **1f** is 100% (no isomer **2f**). ^e^Purity of **1g** is 95% (including isomer **2g** (5%)). ^f^Purity of **1h** is 93% (including isomer **2h** (7%)). ^g^5 mol % of Tf_2_NH was used.

**Scheme 1:**
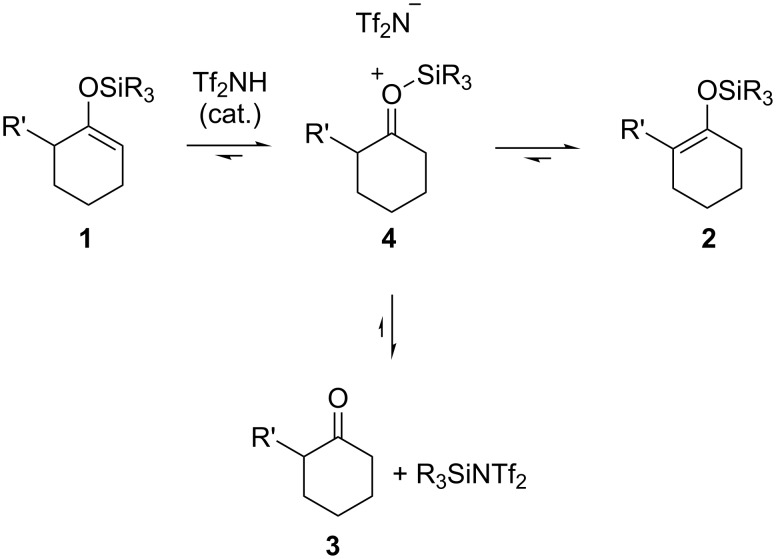
Plausible mechanism for Tf_2_NH-catalyzed isomerization of silyl enol ethers.

A plausible mechanism for the catalytic isomerization is shown in Scheme 1. Silyl enol ether **1** is rapidly protonated by a catalytic amount of Tf_2_NH to give the corresponding siloxonium cation **4**, and, then, another molecule of silyl enol ether **1** deprotonates the α-position of **4**. Equilibration results in the selective production of the thermodynamically more stable **2**. As a side reaction, the counter anion, Tf_2_N^−^, could attack the silicon atom of **2** to produce silyl triflic imide (R_3_SiNTf_2_) [8–12] and the corresponding ketone **3**. Therefore, the use of a large amount of Tf_2_NH causes decomposition into **3** (Table 1, entry 2).

**Scheme 2:**
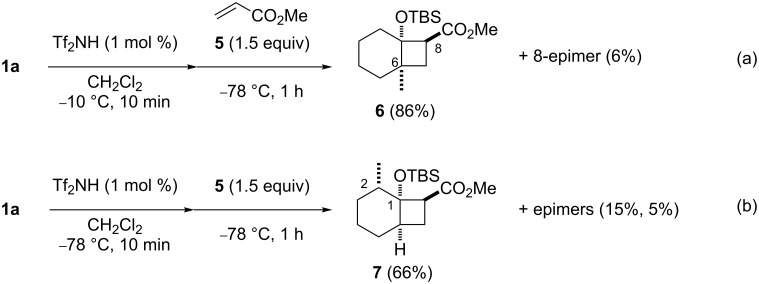
Regioselective formation of bicyclo[4.2.0]octanes from the same substrates by the isomerization–(2 + 2) cycloaddition procedure.

We have previously reported the Tf_2_NH catalyzed (2 + 2) cycloaddition of silyl enol ethers with acrylates generating substituted cyclobutanes [10]. We are intrigued that the isomerization of silyl enol ethers and successive (2 + 2) cycloaddition could be promoted by Tf_2_NH in a one-pot reaction. When **1a** was treated with Tf_2_NH (1 mol %) under the isomerization conditions (−10 °C), followed by the addition of methyl acrylate (**5**) at −78 °C, 6-methylbicyclo[4.2.0]octane **6** and its diastereomer were obtained in 86% and 6%, respectively (Scheme 2a). No formation of their regioisomers was observed. The obtained compound **6** is identical to the product in the reaction of **2a** with **5** [10,13]. It is noteworthy that two different reactions, isomerization and (2 + 2) cycloaddition, are catalyzed by Tf_2_NH [14–18]. By contrast, when **1a** reacted with **5** in the presence of Tf_2_NH at −78 °C, (2 + 2) cycloaddition directly proceeded to give 2-methylbicyclo[4.2.0]octane **7** in 66% yield along with the formation of two diastereomers (Scheme 2b). Obviously, at this temperature, no isomerization of **1a** occurred.

**Scheme 3:**
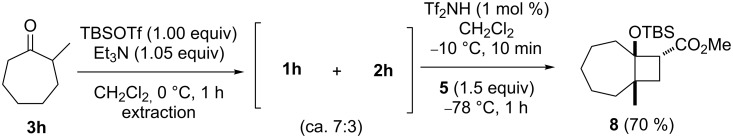
Formation of bicyclo[5.2.0]octane from the regioisomeric mixture of silyl enol ethers.

The above finding can be applied to (2 + 2) cycloaddition, even if a mixture of regioisomeric silyl enol ethers is used as a substrate (Scheme 3). Thus, the reaction of ketone **3h** with TBSOTf in the presence of NEt_3_ afforded a regioisomeric mixture of silyl enol ethers **1h** and **2h** (ca. 7:3). After extraction to remove the amine reagent, the crude regioisomeric mixture was subjected to Tf_2_NH at −10 °C and subsequently reacted with acrylate **5** to afford (2 + 2) cycloadduct **8** in 70% yield. This result indicates that the Tf_2_NH catalyzed reaction can save not only the separation to remove the corresponding kinetically favourable regioisomer, but also loss of the undesired regioisomer.

## Conclusion

In summary, we have developed a new catalytic isomerization reaction of silyl enol ethers. Kinetically favourable silyl enol ethers were smoothly converted into thermodynamically stable ones by treatment with a catalytic amount of Tf_2_NH under mild conditions. Moreover, we demonstrated that the one-pot reaction involves two different catalytic reactions, an isomerization and a (2 + 2) cycloaddition.

## Supporting Information

File 1 Experimental details and spectral data.
