# Supplementary material for: Facile isomerization of silyl enol ethers catalyzed by triflic imide and its application to one-pot isomerization–(2 + 2) cycloaddition
Source: Beilstein J Org Chem. 2012 Apr 27;8:658–61. doi: 10.3762/bjoc.8.73 (PMC3388851; doi:10.3762/bjoc.8.73)
Supplement: File 1 — Experimental details and spectral data. [file Beilstein_J_Org_Chem-08-658-s001.pdf]

Supporting Information

for

Facile isomerization of silyl enol ethers catalyzed by triflic imide and its application to one-pot isomerization–(2 + 2) cycloaddition

Kazato Inanaga[1], Yu Ogawa[2], Yuuki Nagamoto[2], Akihiro Daigaku[1], Hidetoshi Tokuyama[1], Yoshiji Takemoto[2] and Kiyosei Takasu*[2]

Address: [1]Graduate School of Pharmaceutical Sciences, Tohoku University, Aobayama, Sendai 980-8578, Japan and [2]Graduate School of Pharmaceutical Sciences, Kyoto University, Yoshida, Sakyo, Kyoto 606-8501, Japan

Email: Kiyosei Takasu* - kay-t@pharm.kyoto-u.ac.jp

* Corresponding author

Experimental details and spectral data

General Procedure: All nonaqueous reactions were carried out under a positive pressure of argon in dried glassware, unless otherwise noted. Solvents were dried and distilled according to standard protocols. Materials were obtained from commercial suppliers and used without further purification. Column chromatography was performed on Merck silica gel 60 (230–400 mesh), and flash column chromatography was performed on Cica silica gel 60 (spherical/40–100 μm). Preparative thin-layer chromatography (PTLC) was performed on Merck Silica Gel 60 F_{254}, 0.5 mm or 2.0 mm PTLC plates with the indicated eluent. Reactions and chromatography fractions were analyzed by employing precoated silica gel plates (Merck Silica Gel 60 F_{254}). All melting points were measured on a YANACO MP-500P micro melting point apparatus and are uncorrected. IR spectra were measured on a Shimadzu FTIR-8300 spectrometer or JASCO FT/IR-410. The ^{1}H and ^{13}C NMR spectra were recorded on a JEOL AL-400 or JEOL ECP-500 with tetramethylsilane or $CHCl_3$ in $CDCl_3$ as the internal standard. EI mass spectra were recorded on a JEOL JMS-DX-303 or JMS-AX-500 spectrometer. Elemental analyses were performed on YANACO CHN CORDER MT-6 spectrometer. Chemical yields in Table 1 were determined by gas chromatography using a SHIMADZU GCMS 2010.

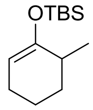

1-*tert*-Butyldimethylsilyloxy-6-methyl-1-cyclohexene (1a) [1].

To a solution of iPr_2NH (1.07 mL, 7.81 mmol) in THF (20.0 mL) was added a 1.69 M hexane solution of BuLi (4.30 mL, 7.27 mmol) at 0 ^{o}C. After stirring was continued for 20 min, to the reaction mixture was added a solution of 2-methylcyclohexanone (0.730 mL, 6.01 mmol) in THF (10.0 mL) −78 °C over 10 min. After 40 min, TBSOTf (1.65 mL, 7.18 mmol) was added, and then the mixture was warmed to rt over 1.5 h. The reaction mixture was quenched with aqueous $NaHCO_3$, extracted with Et_2O twice and washed with brine. The combined organic layers were dried over $MgSO_4$, filtered, and concentrated in vacuo. The residue was purified by column chromatography on silica gel with hexane as eluent, to give silyl enol ether **1a** (990 mg, 73%) as a colorless oil. The spectral data of **1a** were in complete agreement with those of the literature [2]. GC (PEG, 130 °C, 50 kPa): t_R (retention time) = 6.0 min.

Typical procedure for isomerization catalyzed by Tf_2NH (Table 1, entry 3).

To a solution of silyl enol ether **1a** (26.8 mg, 0.118 mmol) in CH_2Cl_2 (1.18 mL, 0.1 M) was added a 0.02 M toluene solution of Tf_2NH (59 μl, 1.18 μmol) at −10 °C. After stirring was continued for 5 min, the reaction mixture was quenched with NEt_3. The conversion yield of **2a** was determined by gas chromatography.

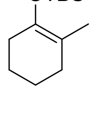

1-*tert*-Butyldimethylsilyloxy-2-methyl-1-cyclohexene (2a).

The spectral data of **2a** were in complete agreement with those reported in the literature [2]. GC

(PEG, 130 °C, 50 kPa): t_R = 6.9 min.

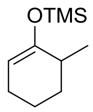

1-Trimethylsilyloxy-6-methyl-1-cyclohexene (1b).

The spectral data of **1b** were in complete agreement with those reported in the literature [3]. GC (PEG, 130 °C, 50 kPa): t_R = 4.7 min.

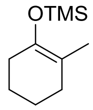

1-Trimethylsilyloxy-2-methyl-1-cyclohexene (2b).

The spectral data of **2b** were in complete agreement with those reported in the literature [4]. GC (PEG, 130 °C, 50 kPa): t_R = 5.0 min.

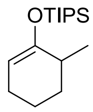

1-Triisopropylsilyloxy-6-methyl-1-cyclohexene (1d).

The spectral data of **1d** were in complete agreement with those reported in the literature [5]. GC (PEG, 130 °C, 50 kPa): t_R = 12.5 min.

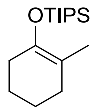

1-Triisopropylsilyloxy-2-methyl-1-cyclohexene (2d).

The spectral data of **2d** were in complete agreement with those reported in the literature [5]. GC (PEG, 130 °C, 50 kPa): t_R = 17.9 min.

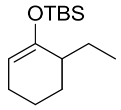

1-*tert*-Butyldimethylsilyloxy-6-ethyl-1-cyclohexene (1e).

Colorless oil; IR (neat) 2858, 1661, 1173, 908, 837 cm^{-1}; ^{1}H NMR (400 MHz) δ 4.78 (t, J = 6.0 Hz, 1H), 1.93 (m, 3H), 1.70 (m, 2H), 1.54 (m, 1H), 1.43 (m, 2H), 1.25 (m, 1H), 0.90 (s, 9H), 0.86 (t, J = 8.0 Hz, 3H), 0.11 (s, 6H); ^{13}C NMR (100 MHz) δ 103.7, 40.4, 27.7, 27.4, 24.8, 24.2, 20.3, 18.1, 11.5, −4.5; LRMS *m*/*z* 240 (M^+); HRMS calcd for $C_{14}H_{28}OSi$: 240.1909, found: 240.1900; GC (PEG, 130 °C, 50 kPa): t_R = 6.8 min.

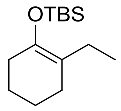

1-*tert*-Butyldimethylsilyloxy-2-ethyl-1-cyclohexene (2e).

Colorless oil; IR (neat) 2858, 1682, 920, 835, 777 cm^{-1}; ^{1}H NMR (400 MHz) δ 2.05 (q, J = 7.8 Hz, 2H), 2.01 (m, 2H), 1.94 (m, 2H), 1.61 (m, 2H), 1.52 (m, 2H), 0.92 (s, 9H), 0.90 (t, J = 7.5 Hz, 3H), 0.09 (s, 6H); ^{13}C NMR (100 MHz) δ 142.3, 117.1, 30.5, 27.7, 27.2, 25.7, 23.8, 23.0, 18.2, 12.3, −3.8; LRMS *m*/*z* 240 (M^+); HRMS calcd for $C_{14}H_{28}OSi$: 240.1909, found: 240.1911; GC (PEG, 130 °C, 50 kPa): t_R = 7.6 min.

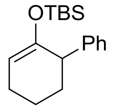

1-*tert*-Butyldimethylsilyloxy-6-phenyl-1-cyclohexene (1f).

The spectral data of **1f** were in complete agreement with those reported in the literature [2]. GC (PEG, 130 kPa): t_R = 13.9 min.

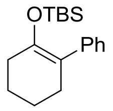

1-*tert*-Butyldimethylsilyloxy-2-phenyl-1-cyclohexene (2f).

The spectral data of **2f** were in complete agreement with those reported in the literature [2]. GC (PEG, 130 kPa): t_R = 18.0 min.

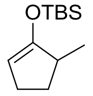

1-*tert*-Butyldimethylsilyloxy-5-methyl-1-cyclopentene (1g).

The spectral data of **1g** were in complete agreement with those reported in the literature [2]. GC (PEG, 130 °C, 50 kPa): t_R = 5.1 min.

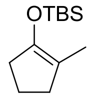

1-*tert*-Butyldimethylsilyloxy-2-methyl-1-cyclopentene (2g).

The spectral data of **2g** were in complete agreement with those reported in the literature [2]. GC (PEG, 130 °C, 50 kPa): t_R = 5.6 min.

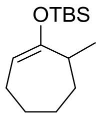

1-*tert*-Butyldimethylsilyloxy-7-methyl-1-cycloheptene (1h).

Colorless oil; IR (neat) 2856, 1651, 1225, 839, 777 cm^{-1}; ^{1}H NMR (400 MHz) δ 4.89 (t, *J* = 6.4 Hz ,1H), 2.36 (m, 1H), 1.96 (m, 2H), 1.52 (m, 6H), 1.06 (d, *J* = 7.8 Hz, 3H), 0.89 (s, 9H) 0.11 (s, 6H); ^{13}C NMR (100 MHz) δ 106.6, 106.5, 39.2, 32.0, 27.8, 26.8, 25.8, 24.1, 18.1, 17.8, −4.7; LRMS *m*/*z* 240 (M^+); HRMS calcd for $C_{14}H_{28}OSi$: 240.1909, found: 240.1902; GC (PEG, 130 °C, 50 kPa): t_R = 7.5 min.

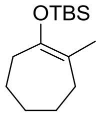

1-*tert*-Butyldimethylsilyloxy-2-methyl-1-cycloheptene (2h).

Colorless oil; IR (neat) 2926, 1676, 1169, 837, 777 cm^{-1}; ^{1}H NMR (400 MHz) δ 2.22 (m, 2H), 1.99 (m, 2H), 1.63 (m, 2H), 1.59 (s, 3H), 1.50 (m, 4H), 0.92 (s, 9H), 0.08 (s, 6H); ^{13}C NMR (100 MHz) δ 133.2, 116.3, 32.8, 31.5, 26.5, 25.9, 25.8, 25.5, 18.8, 18.1, −3.9; LRMS *m*/*z* 240 (M^+); HRMS calcd for $C_{14}H_{28}OSi$: 240.1909, found: 240.1898; GC (PEG, 130 °C, 50 kPa): t_R = 8.2 min.

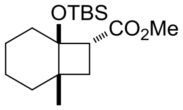

(1*R,6*S**,8*R**)-1-(*tert*-Butyldimethyloxy)-8-(methoxycarbonyl)-6-methylbicyclo[4.2.0]octane (6).**

To a stirred solution of **1a** (45.3 mg, 0.200 mmol) in CH_2Cl_2 (2.0 mL) was added a 0.02 M toluene solution of Tf_2NH (100 μL**,** 2.00 μmol) at −10 °C and stirred for 5 min. After the reaction mixture was cooled to −78 °C, to the resulting solution was added methyl acrylate (**5**; 27.0 μL, 0.300 mmol), and stirring was continued for a further 1 h. The reaction mixture was quenched with saturated $NaHCO_3$ aq and extracted with Et_2O twice. The combined organic layers were washed with brine,

dried over $MgSO_4$, filtered, and concentrated in vacuo. The residue was purified by chromatography on silica gel with hexane–AcOEt (95:5, v/v) as eluent to give **6** (57.4 mg, 86%) as a colorless oil along with its 8-epimer (6%). The spectral data of **6** were in complete agreement with those reported in the literature [6].

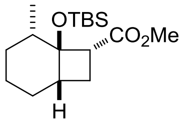

(1*R,2*R**,6*S**,8*R**)-1-(*tert*-Butyldimethyloxy)-8-(methoxycarbonyl)-2-methylbicyclo[4.2.0]octane (7).** To a stirred solution of **1a** (35.8 mg, 0.158 mmol) in CH_2Cl_2 (2.0 mL) was added a 0.02 M toluene solution of Tf_2NH (79 μL, 1.58 μmol) at −78 °C and stirred for 5 min at the same temperature. After the reaction mixture was cooled to −78 °C, to the solution was added **5** (21.0 μL 0.237 mmol), and stirring was continued for a further 2 h. The reaction mixture was quenched with saturated $NaHCO_3$ aq and extracted with Et_2O twice. The combined organic layers were washed with brine, dried over $MgSO_4$, filtered, and concentrated in vacuo. The residue was purified by chromatography on silica gel with hexane–AcOEt (95:5, v/v) as eluent to give **7** (57.4 mg, 66%) as colorless oil along with its epimers (15% and 5%). The stereochemistry of **7** was assigned by coupling constants in ^{1}H NMR. IR (neat) 2928, 1738, 912, 835, 773 cm^{-1}; ^{1}H NMR (400 MHz) δ 3.63 (s, 3H), 2.88 (dd, *J* = 10.4, 2.4 Hz, 1H), 2.33 (m, 1H), 1.90 (tq, *J* = 10.0, 3.2 Hz, 1H), 1.74 (q, *J* = 11.2 Hz, 2H), 1.46 (m, 4H), 1.25 (m, 2H), 0.87 (s, 9H), 0.72 (d, *J* = 6.4 Hz, 3H), 0.18 (s, 3H), 0.15(s, 3H); ^{13}C NMR (100MHz) δ 172.9, 79.7, 51.2, 49.3, 41.6, 33.8, 29.2, 25.7, 23.7, 22.1, 18.6, 18.3, 15.7, −2.5, −2.6; LRMS *m*/*z* 255 (M^+ − 57); HRMS calcd. for $C_{13}H_{23}O_3Si$: 255.1416, found: 255.1423.

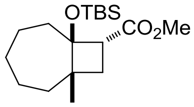

(1*R,7*S**, 9*R**)-1-(*tert*-Butyldimethylsiloxy)-7-methyl-9-methoxycarbonylbicyclo[5.2.0]nonane (8)**.

To a mixture of 2-methylcycloheptanone (589 mg, 4.67 mmol) and Et_3N (683 μL, 4.91 mmol) in CH_2Cl_2 (4.5 mL) was added TBSOTf (1.07 mL, 4.67 mmol) at 0 °C. After being stirred for 1 h at ambient temperature, the resulting mixture was quenched with H_2O and hexane. The aqueous layer was extracted twice with hexane. The combined organic layers were washed with brine, dried with Na_2SO_4, and concentrated in vacuo. To a solution of crude silyl enol ether (**1h**:**2h** = ca. 7:3 determined by ^{1}H NMR) in CH_2Cl_2 (15 mL) was added a solution of Tf_2NH in toluene (0.08 M solution, 2.66 mL, 212 μmol) at −10 °C. After being stirred for 15 min at the same temperature, the resulting mixture was cooled to −78 °C. To the mixture was added **5** (382 μL, 4.25 mmol), and the resulting solution was stirred for 2 h at the same temperature, and then quenched with Et_3N and H_2O. The aqueous layer was extracted twice with Et_2O. The combined organic layers were washed with brine and dried with Na_2SO_4, and concentrated in vacuo. The residue was purified by chromatography on silica gel with hexane–AcOEt (40:1, v/v) as eluent to give **8** (972 mg, 70%, dr > 99:1) as a colorless oil.

IR (neat) 2927, 2856,1739 cm^{-1}; ^{1}H NMR (500 MHz) δ 3.69 (s, 3H), 3.18 (dd, J = 12.2, 12.2 Hz, 1H), 1.82–1.50 (m, 3H), 1.43 (dd, J = 13.8, 10.4 Hz, 1H), 1.21 (m, 2H), 0.92 (s, 9H), 0.20 (s, 3H), 0.08 (s, 3H); ^{13}C NMR (126 MHz) δ 173.3, 82.1, 51.0, 46.9, 45.7, 42.6, 33.7, 31.8, 31.0, 26.1, 24.5, 23.7, 23.5, 18.7, −3.1, −3.2; LRMS m/z 327 (M^+ + 1); Anal. calcd. for $C_{13}H_{34}O_3Si$: C, 66.21; H, 10.49; found: C, 66.42; H, 10.22.

References

1. Takasu, K.; Ishii, T.; Inanaga, K.; Ihara, M. *Org. Synth.* **2006,** *83*, 193–199.
2. Ishihara, K.; Nakamura, H.; Nakamura, S.; Yamamoto, H. *J. Org. Chem.* **1998,** *63*, 6444–6445. doi:10.1021/jo9812936
3. Cazeau, P.; Duboudin, F.; Moulines, F.; Babot, O.; Dunogues, J. *Tetrahedron* **1987,** *43*, 2075–2088. doi:10.1016/S0040-4020(01)86789-2
4. Behenna, D. C.; Stoltz, B. M. *J. Am. Chem. Soc.* **2004,** *126*, 15044–15045. doi:10.1021/ja044812x
5. Magnus, P.; Lacour, J.; Coldham, I.; Mugrage, B.; Bauta, W. B. *Tetrahedron* **1995,** *51*, 11087–11110. doi:10.1016/0040-4020(95)00696-6
6. Takasu, K.; Ueno, M.; Inanaga, K.; Ihara, M. *J. Org. Chem.* **2004,** *69*, 517–521. doi:10.1021/jo034989u
